# A Neural Coordination Strategy for Attachment and Detachment of a Climbing Robot Inspired by Gecko Locomotion

**DOI:** 10.34133/cbsystems.0008

**Published:** 2023-02-24

**Authors:** Bingcheng Wang, Zhouyi Wang, Yifan Song, Weijia Zong, Linghao Zhang, Keju Ji, Poramate Manoonpong, Zhendong Dai

**Affiliations:** ^1^ College of Mechanical and Electrical Engineering, Nanjing University of Aeronautics and Astronautics, Nanjing 210000, China.; ^2^ Nanjing University of Aeronautics and Astronautics Shenzhen Research Institute, Shenzhen 518063, China.; ^3^ Department of Art and Design, Nanjing Tech University, Nanjing 210000, China.

## Abstract

Climbing behavior is a superior motion skill that animals have evolved to obtain a more beneficial position in complex natural environments. Compared to animals, current bionic climbing robots are less agile, stable, and energy-efficient. Further, they locomote at a low speed and have poor adaptation to the substrate. One of the key elements that can improve their locomotion efficiency is the active and flexible feet or toes observed in climbing animals. Inspired by the active attachment–detachment behavior of geckos, a hybrid pneumatic–electric-driven climbing robot with active attachment–detachment bionic flexible feet (toes) was developed. Although the introduction of bionic flexible toes can effectively improve the robot’s adaptability to the environment, it also poses control challenges, specifically, the realization of attachment–detachment behavior by the mechanics of the feet, the realization of hybrid drive control with different response characteristics, and the interlimb collaboration and limb–foot coordination with a hysteresis effect. Through the analysis of geckos’ limbs and foot kinematic behavior during climbing, rhythmic attachment–detachment strategies and coordination behavior between toes and limbs at different inclines were identified. To enable the robot to achieve similar foot attachment–detachment behavior for climbing ability enhancement, we propose a modular neural control framework comprising a central pattern generator module, a post-processing central pattern generation module, a hysteresis delay line module, and an actuator signal conditioning module. Among them, the hysteresis adaptation module helps the bionic flexible toes to achieve variable phase relationships with the motorized joint, thus enabling proper limb-to-foot coordination and interlimb collaboration. The experiments demonstrated that the robot with neural control achieved proper coordination, resulting in a foot with a 285% larger adhesion area than that of a conventional algorithm. In addition, in the plane/arc climbing scenario, the robot with coordination behavior increased by as much as 150%, compared to the incoordinated one owing to its higher adhesion reliability.

## Introduction

Recently, robotics is gradually expanding from traditional industrial fields to a broader range of application scenarios. Thus, it is increasingly crucial for robots to perform autonomously agile movements and dexterous operations in complex unstructured, dynamic environments. However, robots’ autonomous movement and manipulation still face vital issues such as limitations in the structure and drive systems [1], the stability in motion control [2], and acquisition of motion skills [3]. Animals have evolved extraordinary locomotion capabilities to adapt to changes in their environment and meet their needs for feeding, escaping, reproduction, and migration. The structural, perceptual, and neural mechanisms of animal locomotion and the dynamical properties of their complex movements are important sources of inspiration for the design of novel robot structures and improvement of motion control algorithms [4]. Climbing behavior is a superior motion skill that animals have evolved to obtain a more beneficial ecological position for survival in complex natural environments. In contrast, the flexibility, stability, and energy efficiency of current bionic climbing robots fall short of those of animals [5–7]. Robots are characterized by low speed and poor adaptation to attachment substrates, and the work to improve their performance still faces many challenges such as understanding the animal climbing movements and neural control mechanisms, the design of novel adhesion structure drives, implementation of skillful imitation and control of climbing movements, and precision fabrication of complex bionic components [8,9].

Geckos’ ability to climb various slope flexibly on multiple substrates (rough–smooth, flat–curved), as well as their high robustness to multiple perturbations are based on the functional collaboration in the motor skeletal–muscular system [10–12], the flexible attachment organs (paws/toes) [13], fine-grained attachment structures, and fine-tuned regulation [14,15]. For example, the gecko’s autonomously inward-attached–outward-detached toes can generate substantially higher adhesion forces than those required for locomotion attachment [16]. They are well adapted to the functional load requirements during free movement and attachment (especially in natural environments) [17,18]. The characteristics of the macro-scale projections (shape, size, and orientation) and roughness of the substrate will influence the available contact area for the toe, its curvature and configuration, and the number of effective toes in the foot, which, in turn, affect the adhesion of the toe and foot [19–21]. This suggests that the attachment end of climbing robots and bionic attachment structures (devices) should have a distributed structure with active attachment and detachment capabilities [22,23].

We developed an air-driven bionic flexible toe with an active attachment–detachment mechanism for use as the adhesion end of a climbing robot, which has good flexibility and adaptability to smooth plane/arc surfaces [24]. However, its nonlinearity and hysteresis [25] pose challenges for robot control systems. Although multifinger systems [23,26] have excellent control strategies, the proposed robot is driven by both air and electricity, with tandem execution of air-driven flexible paws and electric rigid joints. Thus, it is necessary to determine how to meet the demand for control of the rhythmic attachment–detachment behavior of the end flexible paws and achieve synergistic operation between drive units with different characteristics. This specifically includes control planning of the attachment–detachment behavior according to the mechanical characteristics of the paws, control of hybrid drives with different response characteristics, collaboration of limb-to-limb motion, and compensation of the execution hysteresis between limb and foot.

It has been shown that hybrid-driven systems with air and electric drives can fully exploit the differences between air and electric drive technologies, thereby not only providing excellent large deformation [27], but also improving the stiffness of the actuators, making the mechanisms more maneuverable and applicable. In contrast, the coordination problems caused by the characteristics, such as nonlinearity and hysteresis, of the different drive-actuator units have yet to be solved. For the hysteresis problem of actuators, some studies have proposed solutions from the perspective of time-series coordination. The soft-body pneumatic network developed by Mosadegh et al. [28] enables the playing of piano pieces by controlling the time-series operations of different pneumatic units. Fan et al. [1] proposed a coordination strategy between the joints of a frog-like robot by measuring the response time of its different limbs. In terms of active attachment and release control for climbing, the climbing robot, LEMUR IIB, has an active inward and release attachment actuator with good motion stability and excellent limb motion coordination [29]. However, there is no advantage in its motion speed because it has a similar time hysteresis problem to that of flexible paws during operation. The mechanical properties of the adhesion device are related to the foot-end trajectory planning of the climbing robot, which places demands on the robot’s foot-end contact. For example, to achieve the mechanical properties of the anisotropic adhesive material, the foot-end trajectory of the Stickybot III robot is divided into shear unloading, swing, adhesion preload, shear loading, and grip, resulting in a stable and reliable climbing motion [30]. In the aforementioned robotic studies, the operation of the end-effector is treated as an independent behavior, overlooking the fact that attachment and detachment are synergistic motions of the foot and limb, resulting in low attachment–detachment reliability and limited movement speed of the climbing robot.

The neural system of animal locomotion is a hierarchical, multilevel control system that divides and collaborates [31–33]. The brainstem reticulospinal system excitability is responsible for exerting excitatory stimuli on the lower central system (mainly the central pattern generator [CPG]) to control body orientation and posture. The spinal CPG activates specific motor neurons transmitted to the lower executive system, the skeletal–muscular system, to complete the motor behavior. Introducing the operation of animal neural control circuits into the control system of a robot, resulting in a CPG-based control [34], can significantly enhance the coordination between the moving limbs of the robot [35]. In a previous work, we introduced a modular neural control into the motion control of a wall-climbing robot without an active attachment mechanism [36], which improved its adhesive climbing performance. This showed that although the modular neural control can solve the problem of limb–limb collaboration in adhesive robots, it still needs to be refined to achieve a hybrid-driven control and compensate for the actuation hysteresis.

We focused on the motion coordination problem caused by the combined operation of drive units with different response characteristics in a novel bionic climbing robot, starting from the behavioral rhythm of animal adhesion climbing. In this study, by imitating the rhythmic motion of animal adhesion, we propose a coordination strategy between the flexible toe attachment and climbing robot limbs; test the basic motion parameters of the robot limbs and bionic toes; develop the coordination timing between the nonlinear air drive and robotic electric limb drive; test the coordination performance between the flexible toes, limb joints, and robot’s motion function components; and realize the coordinated motion control of the bionic climbing robot with multiple drive units.

## Inspiration by Active Attachment and Detachment in Gecko Locomotion

The active attachment–detachment movement of geckos is a necessary step in their climbing movements, which is mainly produced by their adhesion organs (toes), and is closely related to the movement state of the locomotion organs (limbs). The coordination between the adhesion and locomotion organs is one of the keys for studying adhesion–disassociation movements. The temporal and spatial variation in both movements needs to be clarified. To this end, experiments were conducted on the adhesive climbing movements of the *Gekko gecko*.

### Gecko motion experiments

#### Experimental animals and equipment

Experimental giant geckos (*Gekko gecko, Linnaeus*) were housed in a greenhouse with controlled humidity and temperature (26 ± 2 °C). Five geckos (*n* = 5), with a weight of 60.5 ± 5.2 g, a body length of 255.5 ± 12.2 mm, and a tail length of 140.4 ± 8.3 mm (± represents standard deviation), were selected for the experiment. The experiment setup consisted of 3 main components: rotatable motion surfaces and channels, a motion capture system, and a data processing platform, with the motion capture system consisting of 4 high-speed motion capture cameras (Prime 17 W, NaturalPoint, Inc., Corvallis, OR, USA) and the Motive motion capture software (Motive 2.1, NaturalPoint, Inc., Corvallis, OR, USA). The motion surfaces and channels were constructed using acrylic panels and profiles, and the channel width was set to 200 mm to allow for unrestricted movement of the giant geckos. The camera frame rate (frames per second) was set to 250. The experimental procedures were approved by the Jiangsu Provincial Association of Laboratory Animal Science (Jiangsu, China, Approval Document No. 2019-152).

#### Experimental procedure

**Fig. 1.**
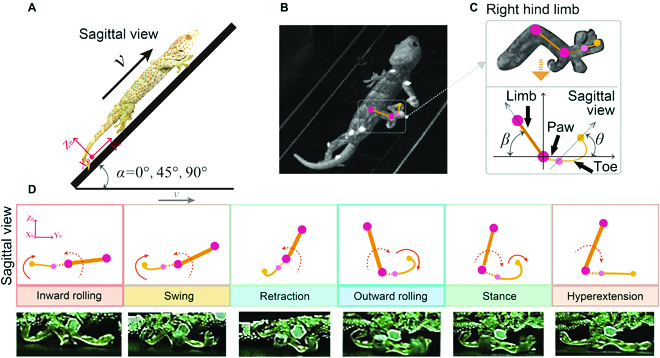
Experiment and parameter definition. (A) *Gekko gecko* performing locomotion tests on substrates at different slope angles, including horizontal, 45° slope, and vertical; (B) video screenshots of *Gekko gecko* moving on a 45° slope plane; (C) parameter definitions of the right hind limb body and pedipalps of *Gekko gecko*; (D) phased demonstration of the attachment–detachment movement cycle.

Prior to the experiment, marker dots were applied to the shoulder/hip, elbow/knee, and wrist/ankle of the geckos, and white nail polish was applied to the heel and tip of the middle toe of their front and back paws as marker dots. The inclination angle *α* of the plane of movement was set to 0°, 45°, and 90°, respectively (Fig. 1A).

#### Parameter definition

The movement cycle of geckos’ limbs can be divided into 2 phases, stance and swing. In the stance phase, the end of the limb (palm of the foot) is in contact with the basal surface of the movement and is mainly responsible for generating thrust. In the swing phase, the end of the limb leaves the basal surface and swings forward in the air. The percentage of time spent in the stance and swing phases of the entire cycle is calculated separately: stance phase (*P_stance_*) and swing phase (*P_swing_*). The toes’ attachment and detachment motion cycle can be divided into 4 phases: hyperextension, inward rolling, retraction, and outward rolling. The hyperextension phase represents the time period when the toes maintain the spread state to keep the adhesion available. The inward rolling phase represents the time period of the inward rolling of the toes and adhesion ability decreases in this state. The retraction phase is the time period when the toes remain retracted to keep the adhesion disabled. The outward rolling phase is the period corresponding to the outward rolling of the toes. The time spent in each of the 4 phases is calculated as a percentage of the overall cycle: *P_he_*, *P_iw_*, *P_rt_*, and *P_ow_* (Fig. 1D). In addition, a coordinate system is established, with the foot wrist/ankle joint as the center. The angles of motion of the foot and limb are defined in the sagittal plane (*YOZ* plane) (Fig. 1B and C): the angle between the plane of the line connecting the toe and heel of the third toe of the foot and the direction of motion (positive direction of the *Y* axis) at the instant of foot contact with the base is defined as the foot contact angle *θ*; the angle between the lower leg and the direction of motion is the contact angle *β*, which can be used as a basis for determining the stance and swing phases.

#### Statistics

SPSS 19.0 (IBM Inc., NY, USA) was used to analyze the selected biological exercise data statistically. The Kruskal–Wallis test and one-way analysis of variance (ANOVA), as well as Scheffe’s method were used to compare the exercise data for different oblique angles (0°, 45°, and 90°), where different oblique angles were the independent variables and the dependent variables included the contact/release time percentage. Welch’s ANOVA and Games–Howell were used for post hoc tests when the chi-squared was not satisfied. A significance level of 0.05 was used. The test results were expressed as mean ± standard deviation.

### Results and analysis of the gecko movement experiment

Timeshare of each phase of attachment–detachment movement: the contact timeshare of the foot was not significantly different across slope and direction of movement (*χ*^2^ = 5.11, *d.f.* = 4,60, *P* = 0.28, K–W), implying that the timeshare required for the foot to make initial contact with the substrate and complete contact with the substrate throughout the foot was close under different movement conditions. The detailed time proportions are listed in Table 1.

**Table 1.** Time proportions of fore and hind foot during different surface movements.

	Proportion (%)							
Foot	*P_rt_*	±SD	*P_ow_*	±SD	*P_he_*	±SD	*P_iw_*	±SD
90°	48.6	10.1	6.4	2.1	28.1	6.6	16.9	6.2
45°	37.2	7.1	7.4	2.4	31.2	4.6	24.4	4.5
0°	33.8	3.8	7.5	1.4	40.8	4.6	17.8	3.7
Limb	*P_swing_*		±SD		*P_stance_*		±SD	
90°	54.05		9.47		46.19		9.34	
45°	44.17		8.4		56.8		8.1	
0°	36.48		6.31		65.68		5.56	

**Fig. 2.**
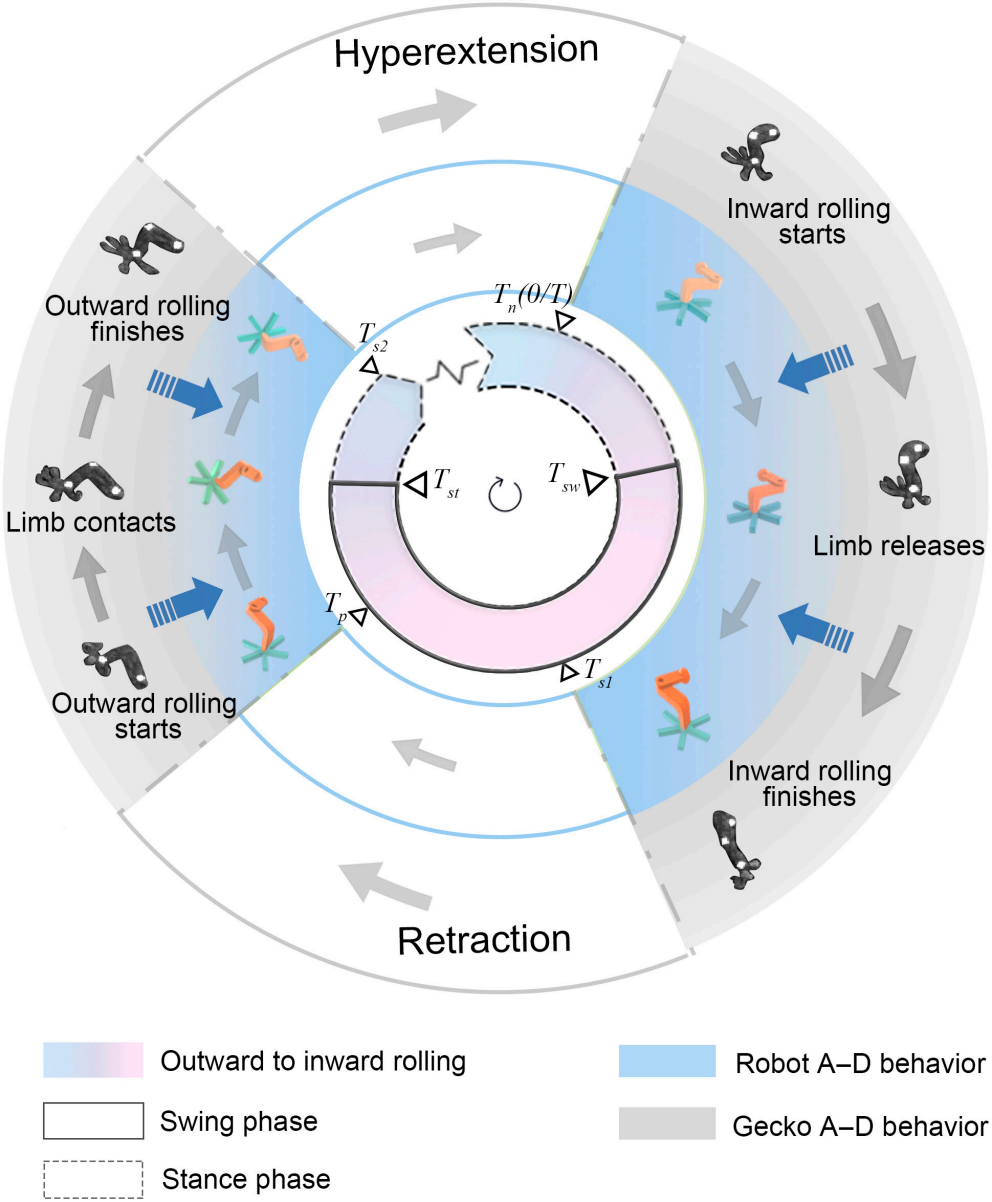
Periodic coordination between the adhesion system and motion system of the gecko and the robot. A–D represents attachment–detachment. This coordination exists in the movement process of the gecko. To apply it to robots, we quantify some key time nodes: *T_i_* represents the time for toes to roll inward, *T*_*s*1_ represents the first time for toes to stop rolling, *T_o_* represents the time for toes to roll outward, *T*_*s*2_ represents the second time for toes to stop rolling. Δ*T*_1_ is from *T_n_* to *T*_*s*1_ and Δ*T*_2_ is from *T_i_* to *T*_*s*2_. The time from *T_n_* to *T*_*s*2_ is fixed, determined by Δ*T*_1_ and Δ*T*_2_, while the time from *T_st_* to *T_o_* can be modified by the motion algorithm.

The results show that the gecko’s adhesion organs (toes) and motion organs (limbs) do not operate in sequence or at the same time. In fact, they have a specific phase difference coordination behavior to help the gecko achieve attachment–detachment. More specifically, in the process of detachment, the motion organ starts to move before the adhesion organ is fully detached. In contrast, the adhesion organ starts to roll outward in the adhesion process before the foot contacts the climbing surface. The specific time matching process is shown in Fig. 2. We reckon that this phenomenon results from the coordinated work of the adhesion and motion organs in time series. The attachment–detachment process of the adhesion organs of geckos and climbing robots requires a specific period of time to reach the desired position, unlike the motion organs, which hardly need additional time. To match them, the following main parameters must be determined:

• The response time Δ*T*_1_, Δ*T*_2_ and the start time *T_n_*, *T_p_* of the attached soles from inward and outward roll;

• The start and end movement time *T_sw_* and *T_st_* of the limb swing phase.

## Bionic Design of Hybrid-Driven Climbing Robot

Inspired by statistical results on biological behavior, we have summarized a temporal phase-based coordination strategy for the robot’s feet and limbs, which is closely related to the hardware and software of the robot. First, we introduce the robot hardware platform.

There are many reasons for the gecko’s extreme athleticism, with the paws and toes being crucial. Geckos’ toes have surface adaptability, which can make them fit the climbing surface in a wide range. More importantly, its active attachment–detachment ability makes for stable motion. However, it is difficult for robots to achieve such behavior, owing to 2 main reasons: (a) the lack of an active attachment–detachment mechanism at the foot end of the robot; (b) the absence of correlation between the attachment–detachment behavior and foot movement behavior.

**Fig. 3.**
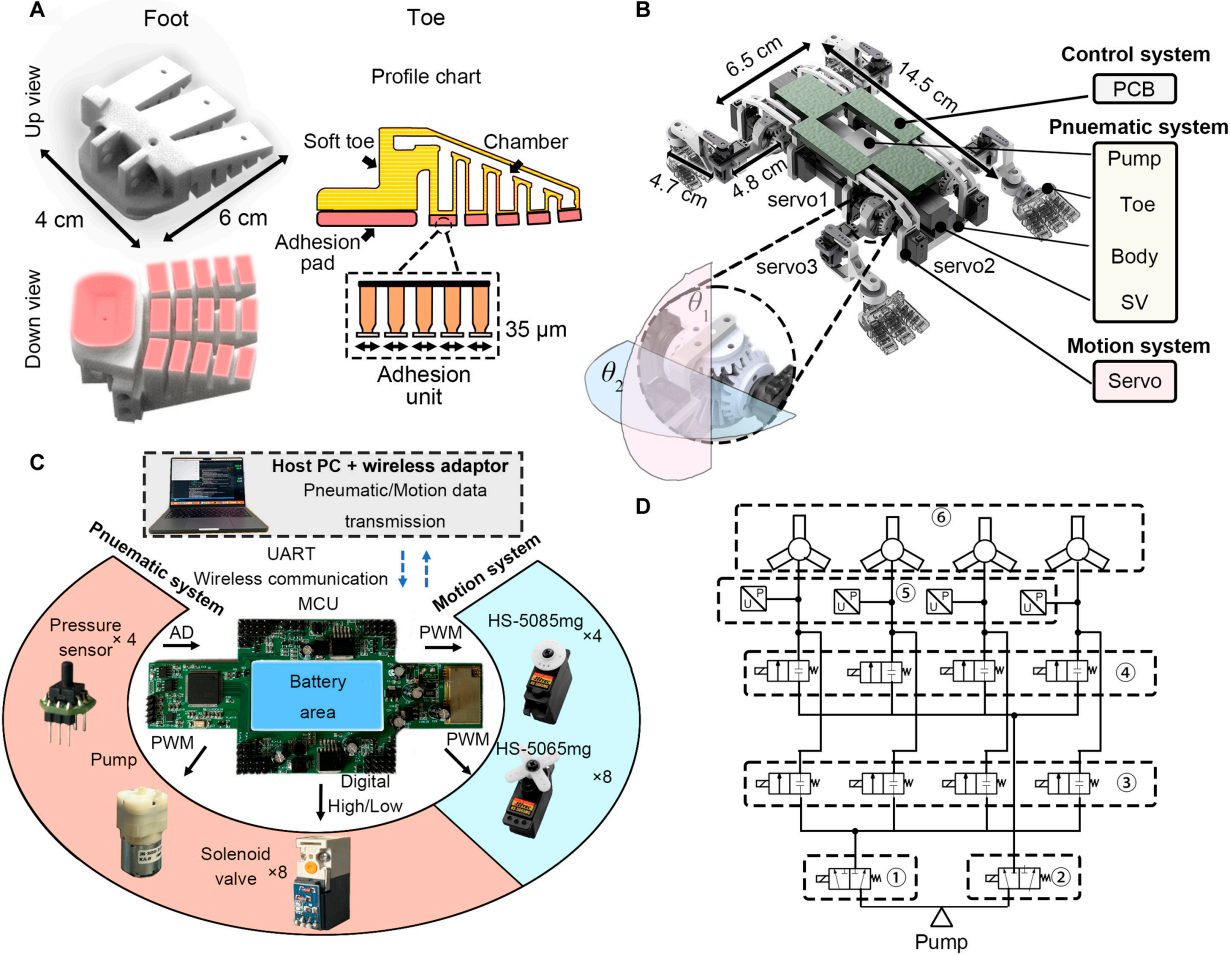
(A) Positive/negative pressure in the bionic adhesion feet is the power source to realize behaviors similar to gecko toe inward/outward rolling through which the robot can realize attachment and detachment. In addition, the toes can deform with different curvatures to adapt to the curved surface. (B) Structural view of the gecko-inspired robot. *θ*_1_, *θ*_2_, and *θ*_3_ represent the angle of hip joint lifting, hip joint stepping, and knee joint stepping, respectively, and their working ranges were −45° to 90°, −35° to 35°, and −90° to 90°, respectively. (C) Composition of robot electronic hardware. (D) Schematic diagram of the pneumatic machine system. ① Main solenoid valves (SV) of the positive pressure pneumatic path, ② main SV of the negative pressure pneumatic path, ③ independent SV group of the positive pressure of the adhesion feet, ④ independent SV group of the negative pressure of the adhesion feet, ⑤ pressure sensor of the adhesion feet, and ⑥ adhesion feet. When the foot works in the positive pressure state, SV1 is connected to the pump and SV2 is connected to the external air. Meanwhile, the SVs in group 3 are open, and the SVs in group 4 are closed. When the foot works in the sealed state, SV1 and SV2 were both connected to the pump, and the SVs in groups 3 and 4 were closed.

We designed an adhesive foot with a soft pneumatic gripper to realize fast bidirectional driving ability and surface adaptability. Three toes were distributed across the robot’s foot end, as shown in Fig. 3A. The bottom layer possesses adhesive material. The manufacturing process of the foot was presented in a previous work [24].

Further, a hybrid-driven robot was designed to combine motor control and pneumatic adhesion control. The structural rendering view is shown in Fig. 3B.

A single air pump scheme was adopted for the pressure source of the pneumatic system of the robot. The working state of the positive/negative pneumatic path was switched through the on-off control of the positive/negative pressure solenoid valves (① and ② in Fig. 3D). Each foot was equipped with 2 positive/negative pressure solenoid valves to set the its positive-pressure/sealing/negative-pressure state. The position near the foot air circuit was equipped with air pressure sensors for air pressure feedback control. Independent positive and negative pneumatic paths were arranged inside the robot fuselage, made by 3-dimensional (3D) printing, for the gas path connection of the pump, SV, the pressure sensors, and the foot. This design ensured the fuselage’s reliability and the gas path’s tightness.

The degree of freedom (DOF) configuration is depicted in Fig. 3B, and the single limb was equipped with 3 active DOFs, which are provided by 3 servos (the angles of these servos are *S*_1_, *S*_2_, and *S*_3_, respectively), and a passive DOF on adhesion foot, which is provided by a bearing. Servo1 and servo2 provide 2 DOFs of hip lifting and swinging joint leg through differential gears. The structure can distribute the resultant moment of leg lifting and walk to servo1 and servo2. Servo3 provides the knee’s DOF. The mapping relationship between motion planning rotation angle and servo angle is shown in Eq. 1:

$$\left[\begin{array}{c} S_{1}\\ S_{2}\\ S_{3} \end{array}]=[\begin{array}{ccc} 1 & 1 & 0\\ 1 & -1 & 0\\ 0 & 0 & 1 \end{array}][\begin{array}{c} \theta_{1}\\ \theta_{2}\\ \theta_{3} \end{array}\right]$$ (1)

To ensure the real-time performance of the sensing and motion system, we designed a printed circuit board (PCB) with high performance and low power consumption, with an STM32H743VIT6 as the main controller, as shown in Fig. 3C. The pneumatic and motion control systems were designed on the PCB for synchronous real-time control. The pressure sensors (CFsensor-XGZP6857A) were designed for measuring the pressure in each pneumatic foot, and the pump (Zhirong Huaguan-ZQ520-02PM) was driven by a MOSFET. Thus the voltage of the pump was determined by the duty factor of the trigger signal, with which the pressure in the foot could be regulated. The state of solenoid valves (OST-T10) determined the on–off of the gas path of each foot. A personal computer was used as the man–machine interface to receive the real-time pressure data and set the modulatory input (MI) in the controller (details in the “CPG-based neural coordination strategy for hybrid-driven motion” section). The PCB control board was on the uppermost layer of the robot with a central hole for a 7.4-V battery providing the energy for all the components.

In total, the robot consisted of 12 electric DOFs and 4 pneumatic DOFs. The fuselage size of the robot was 145 mm × 65 mm × 60 mm, and the mass was 756 g.

## Modular Neural Control for the Hybrid-Driven System

### Electronic hardware setup and the pneumatic control system

**Fig. 4.**
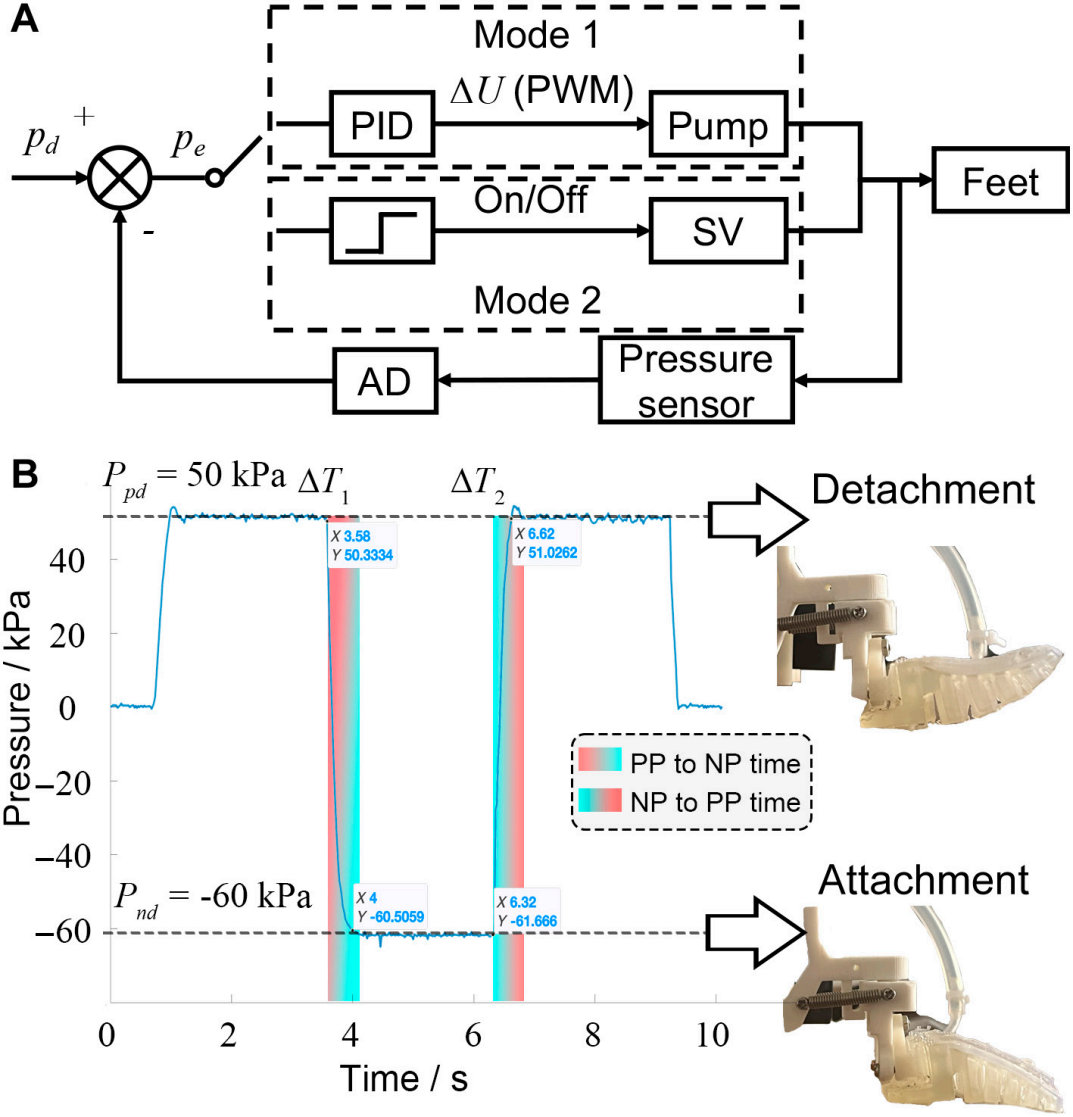
(A) Control diagram of the pressure system. (B) Response time of the pneumatic system. Δ*T*_1_ represents the time from positive pressure (PP) to negative pressure (NP), and Δ*T*_2_ represents the time from negative pressure (NP) to positive pressure (PP).

The hysteresis characteristics of the pneumatic feet are a key factor in the coordination of the robot’s legs and feet, and are closely related to the foot structure and control methods. In this study, we adopted 2 control modes to complement each other, as shown in Fig. 4A. In Mode 1, a PID controller is adopted. Through the adjustment of the pulse width modulated duty cycle, the air pressure of the foot shape is effectively reduced and controlled indirectly. This method reduced the energy consumption of the pneumatic system and improved the response speed; however, it did not allow the simultaneous inward or outward rolling of different feet because the voltage of the pump could not meet all the demands of feet at the same time. To solve the problem of Mode 1, we also established Mode 2, which adopted an electromagnetic threshold swtich (the pump operated at full power), forming a control method similar to bang-bang control. However, the response speed of the solenoid valve was slow, which could cause the air pump to stall. Therefore, the mode was only adopted when Mode 1 was not applicable.

The pneumatic system determined Δ*T*_1_ and Δ*T*_2_. The positive/negative pressure switching time was required to measure and determine the coordination time between the robot motion system and pneumatic system. According to the deformation of toes at different pressures, we set 50 kPa and −60 kPa as the expected inward and outward rolling pressure. We conducted a positive/negative pressure control test on the robot. The results from a single limb are shown in Fig. 4B.

Through the statistical analysis of 10 measurement results, Δ*T*_1_ was 0.4 ± 0.04 s and Δ*T*_2_ was 0.3 ± 0.02 s. To ensure sufficient time to reach the desired pressure, Δ*T*_1_ and Δ*T*_2_ were set to 0.4 s and 0.3 s, respectively, as the adhesion and detachment hysteresis times.

### CPG-based neural coordination strategy for hybrid-driven motion

In Fig. 2, a unified cycle and fixed-phase relationship can be observed between the gecko’s adhesion organs and limbs. Based on gecko locomotion pattern, the coordination of attachment–detachment organs and limbs can effectively promote adhesion strength. To mimic similar periodic active attachment/detachment function and coordination on the robot, a neural coordination strategy (NCS) based on a modular neural control was applied as the hybrid-driven control system.

**Fig. 5.**
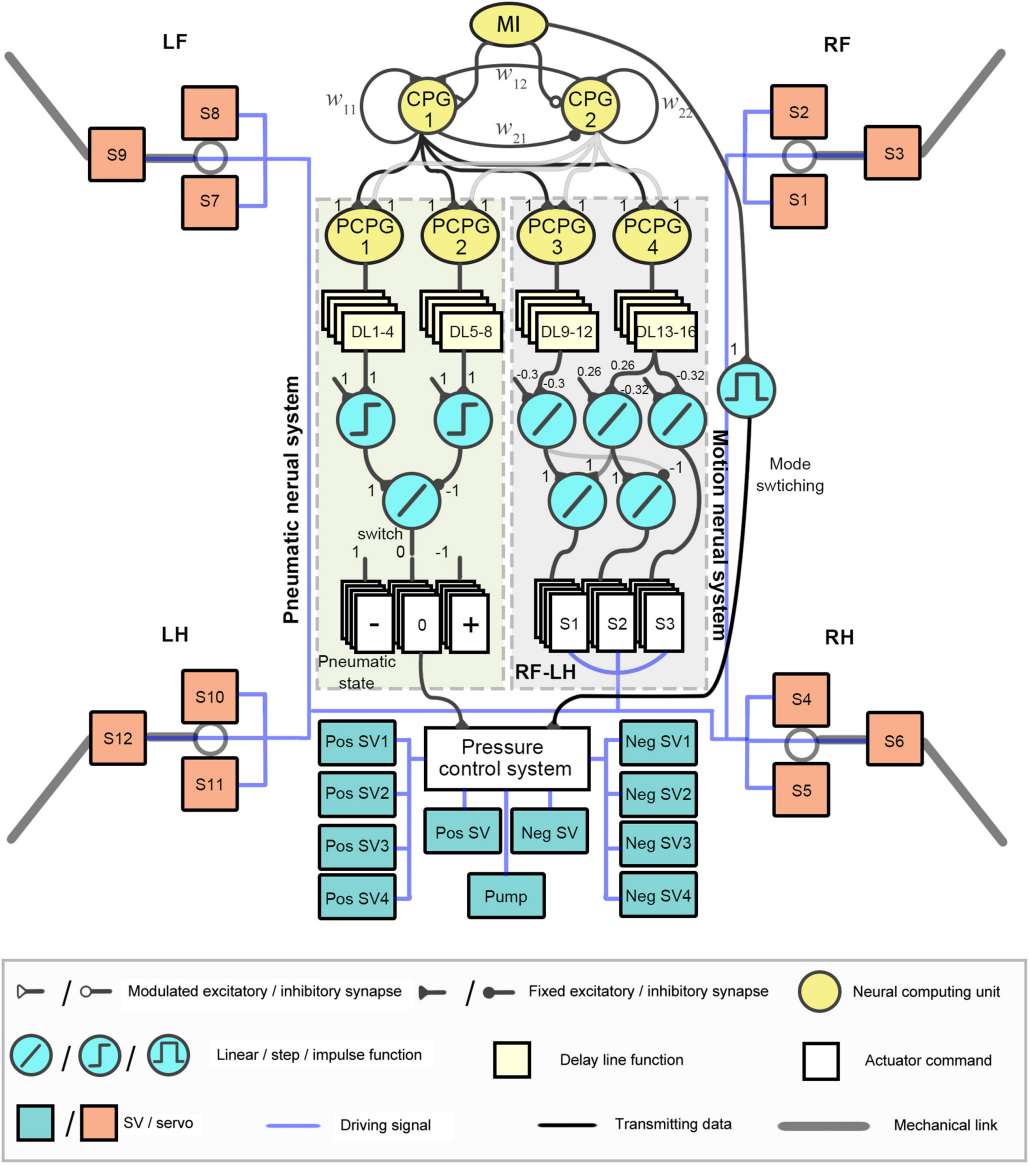
Components of the modular neural control and output correspondence with actuators.

As shown in Fig. 5, the control framework of the NCS adopted in this study was composed of the following 4 parts: (a) CPG module, a rhythm generator with neural characteristics and adjustable frequency; (b) postprocessing of CPG (PCPG) module, which converted the CPG signal into a signal matching to the robot motion; (c) delay line (DL) module, a function to form the coordination between the adhesion and motion organs and the phase relationship between the limbs; and (d) signal mapping module, which adjusted the signal of the PCPG, according to the mechanical/electronic properties of the components and sent it to the actuator.

**Fig. 6.**
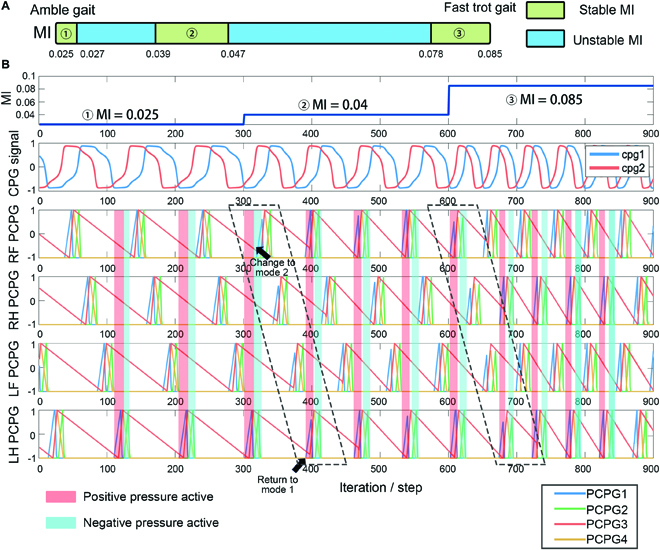
Signals of MI, CPG, and PCPG with DL. RF, RH, LF, and LH represent the right fore limb, right hind limb, left fore limb, and left hind limb, respectively. In the design of the PCPG and DL modules, to reduce the use of Mode 2 for the pressure control system, it was necessary to reduce the conflict between the negative and positive pressure period. It was also necessary to avoid the unstable MI in (A) as it could necessitate the continuous use of Mode 2. Further, there may be a potential risk of damage to the pump. When MI changed in a stable area, the above conflict cannot be avoided. Thus, for the very first time after MI changes, Mode 2 is adopted (time in the black wireframe in (B)). Subsequently after that, Mode 1 took effect again.

In this neural control framework, the CPG module adopts 2 discrete recurrent neurons to generate the basic rhythm signals of periodic oscillation. Through the frequency-adjustable CPG signals, the rhythmic behavior of the robot is formed. Its mathematical expression is shown in Eq. 2:

$$\begin{array}{c} C_{1}(t)=\tanh(w_{11}C_{1}(t-1)+(w_{12}+\mathrm{MI})C_{2}(t-1))\\ C_{2}(t)=\tanh(w_{22}C_{2}(t-1)+(w_{21}-\mathrm{MI})C_{1}(t-1)) \end{array}$$ (2)

where *C*_1, 2_(*t*) represents the output of CPG 1 and 2 at time *t*. *w*_11_, *w*_12_, *w*_21_, and *w*_22_ are the synaptic weights of the network, which were set to 1.4, 0.18, −0.18, and 1.4, respectively, based on our previous work [36]. The MI is used as a frequency regulator, which can realize the continuous adjustment of the gait frequency and pattern (even if MI has an instant change). The CPG output with varied MI is shown in Fig. 6. The MI values of the typical amble (the number of limbs in the stance phase ≥3 at any time) and fast trot (the number of limbs in the stance phase ≥2 at any time) gait were 0.025 and 0.085, respectively. The proposed controller generated these discrete CPG signals at a variable frequency (6 to 25 Hz).

The PCPG was a signal processing unit that divided the rhythm signal into different actuator driving signals. To generate appropriate PCPG signals that were with the actuators, it was necessary to adjust the rise and fall times of the PCPG. In the neural control system, a discrete comparison event was defined as follows, used to record 2 CPGs’ signal states and discrete comparison event *D*(*t*) as (Eq. 3):

$$D(t)=\{\begin{array}{cc} 1 & C_{1}(t)\geq 0\;\text{and}\;C_{2}(t)\leq \mu_{0}\\ -1 & \text{else} \end{array}$$ (3)

where *μ*_0_ determines the duration of the *D*(*t*) positive signal. The integral term $I_{D}^{+}(t)$/$I_{D}^{-}(t)$ of *D*(*t*) was used to characterize the current duration of the positive/negative signal of *D*(*t*), which is (Eq. 4):

$$\begin{array}{c} I_{D}^{+}(t)=\{\begin{array}{cc} I_{D}^{+}(t-1)+D(t) & D(t)\geq 0\\ 0 & \text{else} \end{array}\\ I_{D}^{-}(t)=\{\begin{array}{cc} I_{D}^{-}(t-1)+D(t) & D(t)<0\\ 0 & \text{else} \end{array} \end{array}$$ (4)

The output of PCPG is shown in Eq. 5:

$$P(t)=\{\begin{array}{cc} \frac{2I_{D}^{+}(t)}{T_{+}}-1.0 & D(t)\geq 0\\ \frac{\mu_{1}I_{D}^{-}(t)}{T_{-}}+1.0 & \text{else} \end{array}$$ (5)

where *T*_+_ and *T*_−_ represent, respectively, the total increase time $I_{D}^{+}(t)$ and decrease time $I_{D}^{-}(t)$; *μ*_1_ determined the decrease slope. By adjusting the values of *μ*_0_ and *μ*_1_, the signal’s rise and fall rates could be controlled to form a signal consistent with the robot’s motion.

As shown in Fig. 6, in the neural control system, 4 PCPGs were adopted: PCPGs 1 and 2 were used as negative and positive pressure triggers in the pneumatic system, respectively. Because the conditioned signal was only used for switching the mode of the pneumatic system, it is only necessary to adjust the sum of rise and fall times. PCPGs 3 and 4 were used as lift and forward signals in the motion system, respectively. In the ambling gait, the time of the stance phase was 3 times that of the swing phase. *μ*_0_ and *μ*_1_ of PCPG 3 were 0.87 and 20, respectively, and *μ*_0_ and *μ*_1_ of PCPG4 were 0.87 and 2, respectively.

A DL is a series of discrete units used for the delay and compensation hysteresis. Setting its delay step *t*_*d*,*i*_ was set such that the stimulation of the *i*th neuron was triggered after *t*_*d*,*i*_ cycles. Therefore, the robot could complete the driving of multiple motion/pneumatic system units in one PCPG. The DL was originally proposed to realize the cooperation between the limbs and generate motion gaits such as fast trot and ambling gait. In the proposed system, 16 DLs were adopted, which could facilitate the coordination of the pneumatic system and motion systems in the same limb. The outputs of the DLs are shown in Fig. 6C, and its specific parameter setting will be expatiated on in the “Coordination for the hybrid-driven system” section.

It is worth noting that a single air pump cannot meet the inward and outward rolling requirements of different feet at the same time. In some intermediate gait (duty factor between fast trot gait and ambling gait) with specific MI, there will be a contradiction between the inward and outward rolling of different feet in Mode 1 of the pressure control; therefore, it was necessary to delimit MI. From the ambling gait to the fast trot gait, the range of the MI was studied to determine the value that would not result in contradiction, as shown in Fig. 6A. In addition, when the MI changed to the phase difference between the legs, there may still be confusion about inflation and deflation in the air circuits of different legs. For example, when the MI changed from 0.025 to 0.04, there was a contradiction between the negative pressure demand of the right fore (RF) limb and positive pressure demand of the left hind (LH) limb. To prevent this problem, during one cycle of CPG operation after MI change, the pressure control system of the robot temporarily switched to Mode 2 and then returned to Mode 1. This method underscores that one of the advantages of the CPG system can be demonstrated. Given that MI will not affect the continuity of the CPG signal, as long as it is adjusted within the feasible range, it will not lead to confusion of the inflation and deflation of different gas paths of the robot.

The signal mapping module was used to map and regulate the signals of the PCPGs into the driving signals of actuators, which varied according to their different mechanical and control characteristics.

The expected signals of the pneumatic control system were 1, 0, and −1, representing positive pressure detachment, sealing, and negative pressure adhesion, respectively. The step function was used as the sampling cutoff of PCPGs 1 and 2, and the difference between them was used as the switching signal of the pneumatic system.

For the motion system, the demand signal is the joint angle. The linear relationship between the PCPG and joint angle was established in our previous study [36]. The bevel gear design was adopted for the robot hip; therefore, the rhythmic signal of the servo was obtained based on Eq. 1.

### Coordination for the hybrid-driven system

Over long-term evolution, geckos’ toes have achieved extremely fast movement speed, and geckos can complete the adhesion and detachment behavior in as fast as 15 to 20 ms. However, the robots’ pneumatic toes need 400 ms and 300 ms to complete the adhesion and detachment behavior, respectively. Thus, the robot could not directly apply the adhesion and detachment with a speed that matches that of the gecko. After summarizing the behavioral data (shown in Fig. 2) of geckos, we drew the following 2 conclusions:

• Regardless of the speed of adhesion detachment, *T_sw_* is between *T_n_* and *T*_*s*1_, and *T_st_* is between *T_p_* and *T*_*s*2_, but the specific location is uncertain;

• The time from *T*_*S*1_ to *T_p_* is inversely proportional to the attachment and detachment time.

The robot’s movement speed was at its fastest when it was trotting fast, Δ*T*_1_, and the landing time (the time of PCPG3 signal from 1 to −1) was shorter than that of the ambling gait. According to the first conclusion, *T_sw_* must not exceed *T*_*s*1_ and *T_st_* must not exceed *T_p_*. The robot moved at its slowest speed when it was ambling, and the robot’s toe inward/outward rolling takes longer than the diagonal gait. According to the second conclusion, the time from *T*_*s*1_ to *T_p_* should be minimum (it is best when it was closed to zero). Above all, *μ*_0_, *μ*_1_ of each PCPG module and *t*_*d*,*i*_ of DL modules are adjusted, and the final coordination parameters are listed in Table 2.

**Table 2.** Parameters of PCPG and DL modules. Take the order of RF, RH, LF, and LH swing phase sequences as an example.

		**PCPG1**	**PCPG2**	**PCPG3**	**PCPG4**
*μ*_0_		0.87	0.865	0.87	0.87
*μ*_1_		80	80	20	2
*t*_*d*,*i*_	*i* = 1 (RF)	0	14	6	6
	*i* = 2 (RH)	24	38 (24+14)	30	30
	*i* = 3 (LF)	48	62 (48+14)	54	54
	*i* = 4 (LH)	72	86 (72+14)	78	78

## Experimental Validation and Results

To verify the motion ability of the robot in this design, we conducted a series of experiments to compare the robot with NCS to that without NCS. Thus, an incoordinated behavior was introduced as a comparison item. In this case, the inward rolling, limb detachment, toe outward rolling, and limb attachment of a period ran in sequence instead of a simultaneous work. The model control based on position-time [30] was adopted as the control method, and the key time points are presented as follows: *T_n_* is selected as 0, *T_sw_* is selected as $\frac{T}{20}$, *T*_*s*1_ is selected as $\frac{T}{20}$, *T_p_* is selected as $\frac{T}{5}$, *T_st_* is selected as $\frac{T}{5}$, and *T*_*s*2_ is selected as $\frac{T}{4}$. In the experiments, we adopted the adhesion area, robot speed, and posture angle as the criteria for the validation.

### Experiments on the correlation between adhesion status and coordination behavior

**Fig. 7.**
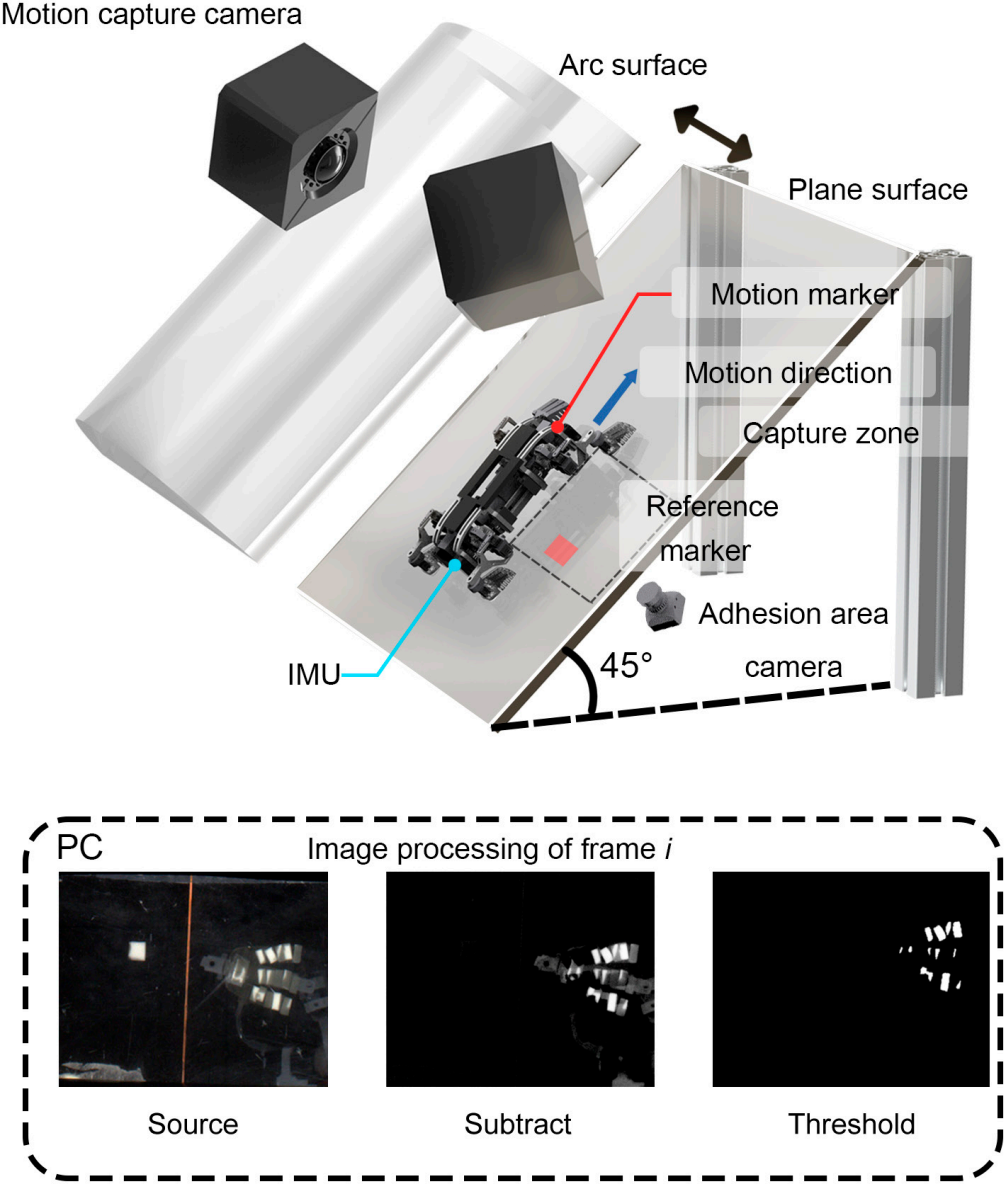
Experimental platforms display. A platform for adhesion area measurement. The camera recorded the adhesion contact throughout the climbing process. To obtain an accurate adhesion area size, the current *i* frame *f_i_* was subtracted from the first frame *f*_1_ (where the robot had not yet made contact with the climbing surface) to eliminate environmental noise and the reference marker. The result was binarized to obtain the total number of pixels of the bright spot *p_i_*. Total number of pixels of the bright spot corresponding to the reference marker was denoted as *p_ref_*. Then, the bright spot adhesion area was $S_{i}=\frac{p_{i}}{p_{\mathrm{ref}}}cm^{2}$. After image processing, the adhesion area of each frame can be obtained, and the maximum value of the adhesion area, which was generated in the adhesion process, during the change in adhesion area could be extracted. This platform was also used as the scene of a robot climbing on an inclined surface. The robot’s spatial coordinates and attitude information during climbing were measured simultaneously.

The first aspect we wanted to demonstrate was whether the coordinated climbing behavior enhanced the adhesion upper limited force during climbing compared to the conventional incoordinated climbing behavior at different overall movement speeds. According to the previous research on geckos’ and soft grippers’ adhesion [37–39], the size of the foot adhesion area is linearly and positively related to the generated adhesion force boundary. Therefore, the upper limit of the adhesion force can be estimated by measuring the size of the adhesion area of the foot. An experimental platform was constructed to obtain the adhesion area generated during the robot climbing processing, as shown in Fig. 7. The climbing surface of the robot is a 50 cm × 30 cm transparent and smooth acrylic flat plate with a strip of light-emmiting diodes around it. With frustrated total internal reflection [40], the adhesion area could be measured when the adhesive material contacted the climbing surface. A camera was attached to the bottom of the test bench to record the pixel area of the spot in the contact area of the single-leg adhesion during the climbing process. A 1 cm ×1 cm marker was attached to the camera area. By comparing the contact spot’s pixel area with the marker’s pixel area, the actual contact area of the foot was calculated. The robot started its movement from one end of the smooth acrylic plate and ran for 3 gait cycles. The robot was subjected to orthogonal experiments based on 3 different overall movement speeds and the presence or absence of coordinated behavior to measure the peak size of the light spot area of the single leg adhesion contact area during each climbing cycle. For the robot parameters, the different overall motion speeds were adjusted by adjusting the CPG generation frequency so that the motion cycles *T* were 4 s, 8 s, and 16 s, respectively, and the robot’s motion cycles were not affected by the presence or absence of coordination behavior. The CPG input MI of the robot was set as 0.025 (ambling gait). To reduce the error caused by chance, each experiment was performed 5 times, with 3 peak points in each experiment. As we mentioned earlier, the adhesion area *S* is linearly and positively related to the upper limit of adhesion force *F_u_*, and we set the adhesion strength coefficient *R*, which indicated the upper limit of adhesion force that could be generated per unit area, which gave us *F_u_* = *RS*(*R* > 0). *R* was mainly influenced by the properties of the adhering material; thus, it can be concluded that *R* was constant in the experiment.

**Fig. 8.**
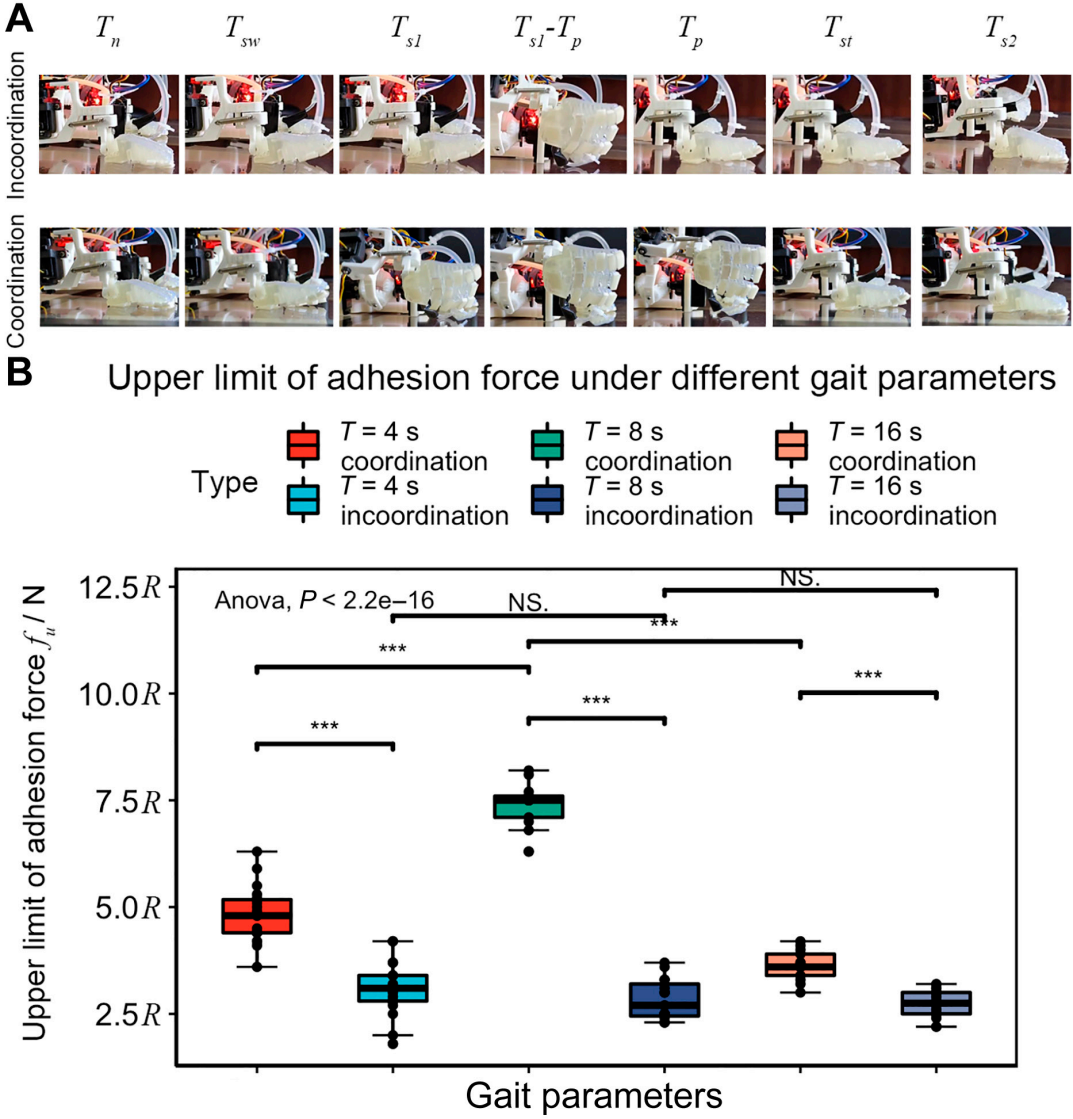
(A) Sagittal view of the robot foot using coordinated and incoordinated control at key moments. (B) Statistical results of upper limit of adhesion force under different control strategies and parameters.

The statistical results of the experiments are shown in Fig. 8. The analysis was completed using R; one-way ANOVA and Tukey HSD tests were used to compare different experimental groups.

Based on the experimental results, there was no significant difference in *F_u_* of all incoordinated movements, with an overall mean of 2.7 cm^2^. In the coordinated movements, *F_u_* tended to increase and then decrease, with the increasing movement period. However, the upper limit was generally higher than the case with incoordinated behavior, and there is a significant difference. The largest upper limit of adhesion force at *T* = 8 s was 7.7*R* cm ^2^ which was 2.85 times larger than the adhesion area for incoordinated motion. This series of results show that the coordinated climbing behavior can effectively improve the adhesion contact state during climbing, compared to the traditional incoordinated climbing behavior. The improvement was most evident at *T* = 8 s.

### Comprehensive climbing ability experiments

**Fig. 9.**
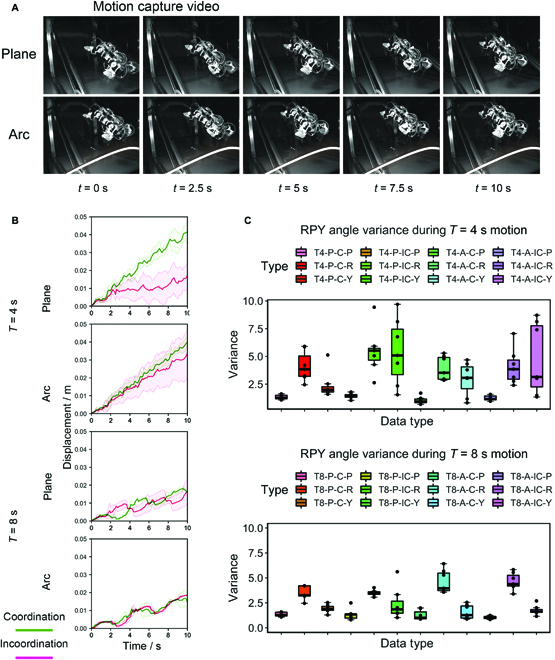
Results of displacement and attitude variance under integrated robot testing. (A) Screenshot of the motion capture; the original video can be found in Movies S1 and S2. (B) Displacement of the robot recorded by the motion capture system, calculated using the coordinates of Cartesian coordinate system. (C) The legend of the attitude sensor consists of 4 bits. The first was the time bit, e.g., T4 for a period of 4 s; the second was the scene bit, P for a plane and A for an arc surface; the third was the strategy bit, C for motion with NCS and IC for motion without NCS; and the fourth was the attitude angle bit.

The climbing speed and vibration of a climbing robot are an important indicator of the stability of the its movement in the current operating environment. This test provided a comprehensive assessment of the climbing performance of the robot with/without coordinated behavior. The robot was tested climbing on an inclined (45°) plane and an arc (50 cm radius of curvature), and the attitude angle and 3D spatial coordinates were recorded during the robot’s movement. The robot’s climbing surface was within the field of view of 2 motion capture cameras (shown in Fig. 7B), which were used to record the position of the robot’s form center in 3D space, and the robot body was equipped with an inertial measurement unit, which was used to record the robot’s current attitude information. The experimental data were recorded for 5 trials. The motion period *T* was set as 4 s and 8 s, and the gait pattern was an ambling gait. The plane/arc video screenshot can be seen in Fig. 9A.

The experiments’ statistical results are shown in Fig. 9.

Figure 9B is the plot of the variation of robot displacement with time. In the experiment with *T* = 4 s, the robot velocity with the NCS was 0.4 cm/s on both the plane and the arc. Meanwhile the average velocity of the robot with incoordinated behavior was 0.16 cm/s on the plane, higher on the arc, averaging 0.32 cm/s. In addition, the standard deviation of the displacement was larger without the NCS. This occurred because the robot slipped during several experiments owing to its unstable motion, whereas the robot with NCS did not slip and thus yielded consistent data. This performance continued in the *T* = 8 s experiment, although the incoordinated motion improved slightly. The stability of the motion on the arc reached the same level as the coordinated motion, with an average speed of 0.2 cm/s. As can be observed, the advantages of the NCS were clear in high-speed motion scenarios where adhesion was more difficult.

Figure 9C shows the variance of the pose angle during robot motion for the combined case. Overall, the difference between using the NCS and not using it was concentrated in the experiments with *T* = 4 s. Owing to the unstable motion of the robot with uncoordinated motion, there were some extensive variance in the pose angle variance data.

## Discussion

In this study, we used an NCS to address the control challenges of hybrid-driven robots: limb-to-limb collaboration and limb-to-foot actuation hysteresis compensation. The design of conventional adhesion-climbing robots reflects a focus on interlimb motor collaboration, which helps the robot balance the forces between legs. In this study, on the other hand, we studied the coordination laws of attachment–detachment behavior of geckos’ toes and paws, applied them to a modular neural control, and establish the NCS. The experimental results presented in the “Comprehensive climbing ability experiments” section demonstrated the improvement in the robot’s climbing stability owing to the NCS. In addition, in terms of speed, the proposed strategy enabled the robot to work with multiple parts simultaneously, reducing the overall operation time, while keeping the operating speed of the moving parts (for our robot, air pumps, motors, etc.) constant.

The most critical aspect was the principle of the mechanism by which the NCS enhanced climbing stability. The main means of stability enhancement include reduction of the adhesion force requirement [41], optimization of the force distribution between the limbs [30], and improvment of the adhesion reliability [42]. In this study, we developed an NCS for a complex system of hybrid-driven climbing robots to improve the adhesion state and meet the adhesion force requirements of large inclination climbing robots. The results of the experiment presented in the “Experiments on the correlation between adhesion status and coordination behavior” section illustrate that the adhesion behavior with the NCS achieved a better adhesion state than that of sequential execution and, more importantly, that different limb contact velocities (reflected in the experiments as different movement cycles, *T* = 4 s, 8 s, and 16 s corresponding to normal contact velocities of 128 mm/s, 64 mm/s, and 32 mm/s, respectively) also affected the adhesion state. The adhesion state was not always determined by the adhesion velocity; rather that the adhesion state was optimal at a particular velocity. In the experiments, this peak occurred at the limb contact velocity corresponding to *T* = 8 s. This result is consistent with those in studies on adhesion capture [43,44]. The NCS enabled the foot to use the limb velocity to aid adhesion, which was achieved with a certain hysteresis in the response of the foot, which corresponds to the first conclusion mentioned in the “Coordination for the hybrid-driven system” section. The traditional sequential execution does not rely on limb velocity for adhesion but only on the toe’s preload to achieve precompression, which is the reason for the lack of variability in the area of adhesion formed by incoordinated strategies in the experiment in the “Experiments on the correlation between adhesion status and coordination behavior” section. Overall, controlled manipulation of the foot (including shape, pressure, attachment, and detachment) was achieved. Although the hysteresis response characteristics of the foot posed some challenges for the control system, together with the neural coordination algorithm, the hysteresis response characteristics were exploited more to improve the adhesion performance of the foot.

Table 3 shows the comparison of the proposed robot and other good climbing robots based on hardware status, core control algorithms, and key performance parameters. Thanks to its flexible feet, the robot successfully adapted to surfaces better than most other climbing robots. In addition, the highly integrated mechanics and control hardware allowed the robot to have a mass of only 756 g, even with 2 drive systems. Generally, climbing robots with active attachment–detachment devices, such as Abigaille-III, are more stable, but are limited by the speed of attachment and detachment, making them relatively slow climbers. However, we successfully increased the speed of the robot to a level similar to that of other robots through the NCS. However, as for the maximum climbing angle, the robot was still not as good as other robots owing to the limited attachment capability of the adhesion foot. Moreover, indicators related to the adhesion capacity also include the robot’s load capacity, which was limited by the adhesion foot, the mass of the robot, and the maximum torque of the servos. NCS could help the robot reach the upper limit of the adhesion capacity of the foot. The robot with NCS had a maximum load of 200 g on the 45° plane, and the one without NCS only had a maximum load of 50 g.

**Table 3.** Hardware status, core control algorithms, and key performance parameters of climbing robots.

	Ours	Slalom[46]	AnyclimbII[47]	Stickybot[48]	Abigaille-III[42]	TBVArobot[49]
Size (cm)	14.5×6.5	35×32	14×12.7	60×20	20×21	10×14
Mass (g)	756	3,100	138	370	635	175
Active A–D	✓				✓	
Power on board	✓	✓		✓	✓	
Neural control	✓	✓				
Model control			✓	✓	✓	
Plane surface climbing	✓	✓	✓	✓	✓	✓
Arc surface climbing	✓		✓			
Force control				✓	✓	
Velocity (body length/s)	0.17	0.29	0.11	0.4	Not given	0.015
Max climbing slope angle	45	30	90	90	90	180

Although our work has significantly improved the robot’s attachment capability, there is still room for improvement in the control algorithm. The limb force distribution in the stance phase, as mentioned before, needs to be optimized. However, the program currently has 2 problems: (a) the proposed scheme relies on the deviation of the actual position of the center of mass (or foot end) from the desired one, leading to a slippage of the center of mass in the robot motion, which affects the speed of the center of mass; (b) although the forces were somewhat evenly distributed on the stance phase limbs, the optimal force pattern still could not be achieved. The online real-time calculation of optimal foot-end forces commonly used in quadruped robots is a theoretically feasible technical solution [45]. However, the model is currently limited by the arithmetic power of the central controller of the climbing robot, which is not yet capable of real-time computing. If the problem of inter-leg force distribution could be solved with low arithmetic power, the robot’s motion’s stability would be significantly improved.

Finally, it is crucial to improve the mechanics. The novel electrically hybrid-driven robot proposed was equipped with flexible pneumatically adherent paws, allowing the robot to adapt to different surfaces (as shown in the “Comprehensive climbing ability experiments” section). However, its response time significantly constrained the robot’s movement speed. Without considering the behavior of the robot’s feet, the robot can move at speeds of up to 2.5 cm/s. In contrast, its maximum movement speed was constrained to 1 cm/s to ensure inward and outward rolling time of the robot’s feet. In addition, the robot’s current mass/length ratio was too large, compared to that of other climbing robots, which affected its maximum climbing angle.

In this study, a novel NCS was proposed to address the problems of the collaboration of limb-to-limb motion and the compensation of execution hysteresis between the limbs and feet of climbing robots. A quantity of behavioral data on the feet and limbs of geckos was recorded and analyzed on different slope angles, and the rhythmic patterns in time sequences were summarized. Inspired by phase differences in different functional organs, we combined a modular neural control framework with adjustable hysteresis adaptation to realize the coordinated behavior of the robot’s limbs and feet. The results of a series of experiments showed that the adhesion area generated using the NCS was 285% higher than that with traditional sequential execution. Moreover, the movement speed increased to 0.4 cm/s on a 45° slope angle, while the original speed was 0.16 cm/s on average. This confirmed that hybrid-driven robots with NCS control had significantly improved operational stability. At present, the biggest drawback of NCS is the lack of the ability to sense the attachment–detachment state and regulate the attachment–detachment behavior. In the future, we will incorporate force sensing systems in the robot’s limbs and further upgrade the NCS to enable stance-phase limb force distribution with low complexity.

## Data Availability

The data used to support the findings of this study are available from the corresponding author upon request.

## Supplementary Materials

Supplementary Materials The supplementary movies show the robot’s motion capacity. Movie S1. Plane 45-degree-slope-angle surface climbing with the NCS. Movie S2. Arc 45-degree-slope-angle surface climbing with the NCS.
